# Tuberculose intestinale révélée par une occlusion intestinale aigüe au cours d’une réaction paradoxale au traitement anti-tuberculeux chez un patient immunocompétent: à propos d’un cas et revue de la littérature

**DOI:** 10.11604/pamj.2019.32.173.17893

**Published:** 2019-04-10

**Authors:** Bruce Wembulua Shinga, Alassane Dièye, Ndèye Méry Dia Badiane, Ndèye Aissatou Lakhe, Viviane Marie-Pierre Cisse Diallo, Khadiatou Diallo Mbaye, Daye Ka, Aboubakar Sidikh Badiane, Assane Diouf, Louise Fortes Déguénonvo, Cheikh Tidiane Ndour, Moussa Seydi

**Affiliations:** 1 Service des Maladies Infectieuses et Tropicales, Centre Hospitalier National Universitaire de Fann, Dakar, Sénégal; 2 UFR Santé, Université Gaston Berger, Saint-Louis, Sénégal

**Keywords:** Tuberculose intestinale, réaction paradoxale, occlusion intestinale aigüe, Intestinal tuberculosis, paradoxical reaction, acute bowel occlusion

## Abstract

La tuberculose intestinale représente 3 à 5% de toutes les localisations viscérales. Malgré l'efficacité démontrée des anti-tuberculeux, des cas d'exacerbation du tableau clinique initial ont été décrits à l'initiation du traitement. Ces réactions dites «paradoxales» sont cependant rarement rapportées chez les immunocompétents et beaucoup moins sous forme d'occlusion intestinale. Nous rapportons un cas de tuberculose intestinale révélée par une occlusion intestinale aigüe au cours d'une réaction paradoxale aux anti-tuberculeux. Il s'agit d'un patient de 26 ans, immunocompétent qui a présenté un syndrome occlusif à un mois de traitement d'une tuberculose pleuro-pulmonaire. La tomodensitométrie (TDM) abdominale était en faveur d'une occlusion intestinale grêlique. La laparotomie objectivait une masse intra-péritonéale avec de multiples adhérences. L'examen anatomopathologique de la pièce opératoire était en faveur d'une tuberculose intestinale. L'évolution était favorable après la poursuite du traitement anti-tuberculeux initial.

## Abstract (en)

Abdominal tuberculosis accounts for 3 to 5% of all visceral diseases. Despite the demonstrated effectiveness of anti-tuberculosis treatments, some cases of exacerbation of the initial clinical presentation have been described during the initiation of treatment. However, these reactions also known as “paradoxical” have been rarely reported in immunocompetent patients and much less in the case of bowel obstruction. We report a case of intestinal tuberculosis revealed by acute bowel obstruction during paradoxical reaction to anti-tuberculosis treatment. The study included a 26-year old immunocompetent patient with occlusive syndrome after a month of treatment for pleuropulmonary tuberculosis. Abdominal computed tomography (CT) showed small bowel obstruction. Laparotomy objectified intraperitoneal mass with multiple adhesions. Anatomo-pathological examination of the surgical specimen showed intestinal tuberculosis. Patient's outcome was favorable after the continuation of initial antituberculosis treatment.

## Introduction

La tuberculose demeure un problème mondial de santé publique. Selon l'organisation mondiale de la santé (OMS) en 2016; 10,4 millions des personnes l'ont contracté avec 1,7 million de décès [1]. Elle est due au *mycobacterium tuberculosis*, une bactérie à fort tropisme pulmonaire. 14% des cas de tuberculose rapportés dans le monde sont extra-pulmonaires [2]. L'atteinte gastro-intestinale représente 3 à 5% de toutes les localisations viscérales. Elle est révélée par une occlusion intestinale dans 20 à 27% des cas [3]. L'efficacité des anti-tuberculeux n'est plus à démontrer. Toutefois, certains cas d'exacerbation du tableau clinique initial ont été rapportés à l'instauration du traitement. Ces réactions dites «paradoxales» et actuellement mieux décrites dans la littérature sont cependant rarement rapportées chez les immunocompétents et beaucoup moins sous forme d'occlusion intestinale [4]. Nous rapportons un cas de tuberculose intestinale révélée par une occlusion intestinale aigüe au cours d'une réaction paradoxale chez un immunocompétent sous anti-tuberculeux.

## Patient et observation

**Figure 1:**
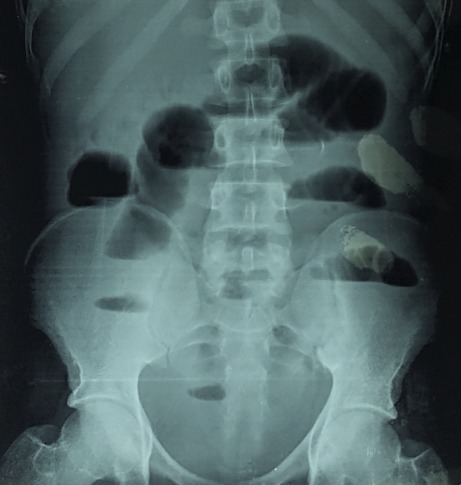
radiographie de l’abdomen sans préparation montrant des niveaux hydo-aériques de type grélique

**Figure 2:**
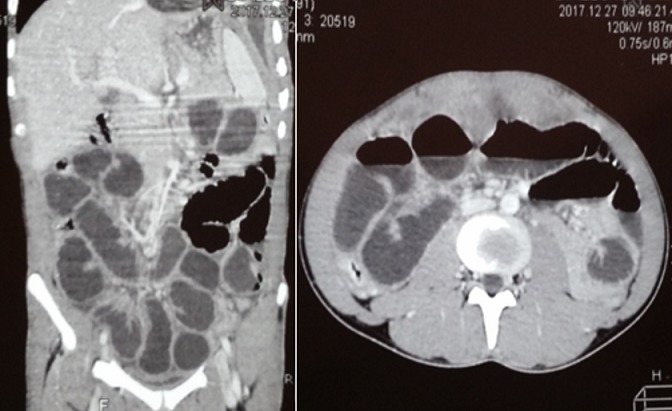
TDM abdominale montrant une dilation d’anses contrastant avec des anses plates et un côlon plat

Il s'agissait d'un patient de 26 ans, sans notion de contage tuberculeux retrouvé, qui avait consulté au service des maladies infectieuses et tropicales (SMIT) de l'hôpital Fann pour une toux chronique, une fièvre vespéro-nocturne et une altération de l'état général. Le début de la symptomatologie remonterait à environ 8 mois, marqué par la survenue d'une toux productive ramenant des expectorations jaunâtres non striées de sang, sans douleur thoracique ni dyspnée. Ce tableau évoluait dans un contexte de fièvre vespéro-nocturne, de sueurs nocturnes sans frissons, d'asthénie physique, d'anorexie non sélective et d'amaigrissement non chiffré. Ce tableau avait motivé plusieurs consultations. Une antibiothérapie non spécifique était prescrite sans amélioration. A son admission au SMIT, l'examen retrouvait: une fièvre à 38,5°C, un syndrome de condensation pulmonaire bilatérale et un syndrome d'épanchement pleural liquidien droit. La NFS avait objectivé une hyperleucocytose à 11.000/mm^3^ avec une CRP positive à 151 mg/l. Les hémocultures étaient négatives, la glycémie à jeun à 0,95 g/l et la sérologie rétrovirale négative. L'analyse du liquide pleural avait objectivé un liquide jaune citrin, exsudatif avec les protides à 40g/l et 320 éléments/mm^3^ 100% lymphocytaire. La bactériologie du liquide pleural était négative de même que le GeneXpert. La radiographie du thorax était en faveur d'une pleuro-pneumopathie droite. La biopsie pleurale n'était pas réalisée. Devant la chronicité du tableau, l'échec de l'antibiothérapie non spécifique et les caractéristiques 'biologiques du liquide pleural, nous avons retenu le diagnostic d'une tuberculose pleuro-pulmonaire. Le patient a été mis sous anti-tuberculeux (2RHZE/4RH). L'évolution a été favorable avec une apyrexie stable et une régression des symptômes pulmonaires. Après un mois de traitement anti-tuberculeux, le patient était revenu en consultation pour une douleur abdominale d'installation progressive, intense à type de colique, diffuse suivie d'un arrêt des matières puis des gaz, accompagnée des vomissements alimentaires puis bilieux et d'un ballonnement abdominal modéré. A l'examen, l'abdomen était ballonné, sensible avec une défense diffuse, tympanique sans bruits hydro-aériques. La radiographie de l'abdomen sans préparation (ASP) mettait en évidence des images hydro-aériques plus larges que hautes de type grêlique Figure 1. La TDM abdominale avait confirmé le syndrome occlusif grêlique avec en plus la présence d'une anse à paroi épaisse, de topographie antéro-médiane encastrée au sein d'un épaississement épiploïque et un épaississement péritonéal pariétal réalisant des foyers de scalopping sur le foie Figure 2. Le patient était référé en chirurgie pour une laparotomie. Le constat per opératoire avait fait état d'une masse intra-péritonéale d'environ 5cm, blanc-grisâtre, ferme à surface irrégulière avec des multiples adhérences grêlo-grêliques et grêlo-pariétales. L'acte opératoire avait consisté à l'ablation de la masse complétée par une adhésiolyse. Les suites opératoires étaient sans particularité. L'examen anatomopathologique de la pièce opératoire avait objectivé une plage de nécrose éosinophile et acellulaire entourée de cellules épithélioïdes et d'une couronne lymphocytaire associée à des cellules géantes de Langerhans sans signe de malignité. Cet aspect histologique était en faveur d'une tuberculose intestinale caséo-folliculaire. Le traitement anti-tuberculeux était poursuivi jusqu'à six mois et le patient avait été déchargé à J5 post opératoire. L'évolution était favorable avec une disparition de tous les symptômes.

## Discussion

Selon les régions, la prévalence de la tuberculose extra-pulmonaire (TBE) variait entre 20 et 25% en 2009 [2]. Actuellement, nous assistons à une relative augmentation des cas. Au Sénégal le nombre des cas de TBE enregistré est passé de 1366 en 2011 à 1618 en 2013, soit une augmentation de 8,1% en 2 ans [5, 6]. Cette augmentation serait due en partie à l'expansion de l'infection à VIH. La tuberculose gastro-intestinale reste cependant, une forme rare de TBE. C'est le 6ème foyer extra-pulmonaire et la principale cause d'occlusion intestinale dans les pays en voie de développement [3, 7]. Un foyer pulmonaire est associé dans 20 à 25% des cas. Dans ce contexte, l'atteinte digestive est d'autant plus fréquente et massive que l'atteinte pulmonaire est sévère [3, 8]. Environ 2 à 23% des patients mis sous anti-tuberculeux connaissent une exacerbation de leur tableau clinique initial et/ou une apparition d'un nouveau foyer actif [4, 9]. Connu depuis 1984, cette réaction « paradoxale » à l'initiation des anti-tuberculeux reste une éventualité rare chez les immunocompétents et exceptionnellement révélée par des signes d'atteinte digestive [9-11]. Quoi que ces derniers soient divers et variés, 75 à 90% des patients se plaignent des douleurs abdominales et d'amaigrissement. Par ailleurs, le tableau clinique peut être d'emblée celui d'une complication à savoir une occlusion intestinale (20% des cas) ou exceptionnellement une perforation viscérale ou une hémorragie digestive [3, 12]. Notre patient était immunocompétent et a présenté une tuberculose intestinale compliquée d'une occlusion intestinale aigüe au cours d'une réaction paradoxale survenue à un mois d'anti-tuberculeux. Le diagnostic de la tuberculose intestinale est difficile vu le caractère peu accessible et pauci-bacillaires des lésions. L'imagerie a une grande valeur d'orientation mais manque de spécificité. La culture sur une pièce de biopsie n'est positive que dans 10 à 20% des cas et nécessite 1 à 2 semaines. La Polymerase Chain Reaction (PCR) quant à elle, permet d'obtenir une réponse rapide mais présente à la fois une faible sensibilité et spécificité pour cette localisation [3, 13]. Kulkarni *et al.* identifient l'examen anatomopathologique comme étant le gold standard pour le diagnostic de la tuberculose intestinale [5].

Chaabane *et al.* décrivent 4 formes macroscopiques des lésions tuberculeuses intestinales: la forme ulcéreuse qui est fréquente sur terrain immunodéprimé, la forme hypertrophique plus rencontré chez les immunocom-pétents, la forme sténosante plus souvent grêlique et la forme ulcéro-hypertrophique qui peut mimer une néoplasie digestive [8]. Chez notre patient, l'aspect macroscopique des lésions intestinales était de type hypertrophique avec des foyers d'épaississement grêlique et épiploïque. L'aspect microscopique de granulome à cellules géantes de type Langhans avec une nécrose caséeuse centrale est très suggestif de la tuberculose. Lorsqu'il est présent et typique, le diagnostic est presque certain [14, 15] comme ce fut le cas chez notre patient. Dans la pratique, la réaction paradoxale est un diagnostic d'exclusion. Toute exacerbation du tableau clinique chez un patient déjà sous anti-tuberculeux doit systématiquement faire rechercher une pathologie intercurrente, une résistance aux anti-tuberculeux ou une inobservance thérapeutique [16]. Une fois retenu, la conduite thérapeutique consiste à maintenir les anti-tuberculeux initiaux. La durée du traitement dépendra donc des foyers actifs présents. Pour ce qui nous concerne, le traitement de la tuberculose digestive est médical en dehors des complications telles que l'occlusion intestinale, la perforation des viscères ou un syndrome hémorragique qui nécessitent un traitement chirurgical [3]. Le Center for Disease Control (CDC) et l'American Thoracic Society (ATS) s'accordent sur un régime thérapeutique identique à la tuberculose pulmonaire à savoir de 6 mois de traitement (2RHZE+4RH) [17, 18]. Notre patient était observant et n'avait aucun argument en faveur d'une pathologie intercurrente. La culture ou le GeneXpert de la pièce biopsique n'étant pas réalisé chez notre patient, nous ne pouvions pas à priori, exclure formellement la possibilité d'une résistance aux anti-tuberculeux instaurés. Toutefois, la bonne évolution sous le même traitement anti-tuberculeux constitue un argument indirect de taille. Lee *et al.* ont rapporté un cas similaire de réaction paradoxale révélé par une sub-occlusion intestinale chez un immunocompétent à 3 mois d'anti-tuberculeux pour une tuberculose pulmonaire. Dans cette observation, le patient avait subi une chirurgie digestive et avait bien évolué sous le même traitement anti-tuberculeux initial [11]. Ha *et al.* ont par ailleurs rapporté que la survenue d'une occlusion intestinale sous anti-tuberculeux pourrait s'expliquer par une fibrose cicatricielle causée par un foyer intestinal méconnu mais présent dès l'initiation du traitement [12]. Notre patient n'avait présenté aucun signe digestif à l'initiation du traitement. Toutefois, nous ne pouvons pas exclure formellement l'existence d'une atteinte digestive concomitante au foyer pulmonaire initial qui se serait secondairement compliquée d'occlusion intestinale sur brides adhérentielles. Ainsi cette hypothèse reste envisageable en dehors de la réaction paradoxale.

## Conclusion

L'atteinte intestinale est la 6^ème^ forme extra-pulmonaire de la tuberculose et la principale cause d'occlusion intestinale dans les pays en voie de développement. Son apparition au cours d'une réaction paradoxale aux anti-tuberculeux bien que rare reste possible, même chez un immunocompétent. Toutefois, elle reste un diagnostic d'exclusion.

## Conflits d’intérêts

Les auteurs ne déclarent aucun conflit d'intérêts.
